# Immune Response to *Rotavirus* and Gluten Sensitivity

**DOI:** 10.1155/2018/9419204

**Published:** 2018-03-15

**Authors:** Antonio Puccetti, Daniele Saverino, Roberta Opri, Oretta Gabrielli, Giovanna Zanoni, Andrea Pelosi, Piera Filomena Fiore, Francesca Moretta, Claudio Lunardi, Marzia Dolcino

**Affiliations:** ^1^Immunology Area, Pediatric Hospital Bambino Gesù, Viale San Paolo 15, 00146 Rome, Italy; ^2^Department of Experimental Medicine, Section of Histology, University of Genova, Via G.B. Marsano 10, 16132 Genova, Italy; ^3^Department of Experimental Medicine, Section of Human Anatomy, University of Genova, Via De Toni 14, 16132 Genova, Italy; ^4^Immunology Unit, University Hospital of Verona, Piazzale L.A. Scuro 10, 37134 Verona, Italy; ^5^Department of Medicine, University of Verona, Piazzale L.A. Scuro 10, 37134 Verona, Italy

## Abstract

*Rotavirus* is a double-stranded RNA virus belonging to the family of *Reoviridae*. The virus is transmitted by the faecal-oral route and infects intestinal cells causing gastroenteritis. Rotaviruses are the main cause of severe acute diarrhoea in children less than 5 years of age worldwide. In our previous work we have shown a link between rotavirus infection and celiac disease. Nonceliac gluten sensitivity (NCGS) is emerging as new clinical entity lacking specific diagnostic biomarkers which has been reported to occur in 6–10% of the population. Clinical manifestations include gastrointestinal and/or extraintestinal symptoms which recede with gluten withdrawal. The pathogenesis of the disease is still unknown. Aim of this work is to clarify some aspects of its pathogenesis using a gene array approach. Our results suggest that NCGS may have an autoimmune origin. This is based both on gene expression data (i.e., TH17-interferon signatures) and on the presence of TH17 cells and of serological markers of autoimmunity in NCGS. Our results also indicate a possible involvement of *rotavirus* infection in the pathogenesis of nonceliac gluten sensitivity similarly to what we have previously shown in celiac disease.

## 1. Introduction

Nonceliac gluten sensitivity (NCGS) can be defined as a nonallergic condition in which the consumption of gluten can lead to symptoms similar to those observed in celiac disease (CD). NCGS is characterized by the absence of celiac specific antibodies (against tissue transglutaminase, endomysium, and/or deamidated gliadin peptide) and absence of classical enteropathy (Marsh 0-1) although an increased density of CD3+ intraepithelial lymphocytes can be observed in duodenal biopsies. Patients with NCGS may have variable HLA status, and positivity for HLA-DQ2 and/or DQ8 has been found in roughly 50% of patients with NCGS. Serological analyses of NCGS patients revealed a high prevalence (40–50%) of first generation antigliadin IgG antibodies. NCGS is characterized by symptoms that usually occur soon after gluten ingestion and disappear or improve with gluten withdrawal but relapse following gluten challenge. The clinical presentation of NCGS may be a combination of gastrointestinal symptoms, including abdominal pain, bloating, bowel habit abnormalities (diarrhoea or constipation), and systemic manifestations, that is “foggy mind,” fatigue, muscle and joint pain, leg or arm numbness, eczema and skin rash, depression, and anemia. Similarly to patients with CD, subjects with clinical manifestations compatible with NCGS should start a gluten-free diet. Since it is still not clear whether NCGS is a permanent or transient condition, reintroduction of gluten after 1-2 years on a gluten-free diet can be considered [1, 2].

Rotavirus is a double-stranded RNA virus belonging to the family of *Reoviridae.*

The virus is transmitted by the faecal-oral route and infects intestinal cells causing gastroenteritis. Rotaviruses are the main cause of severe acute diarrhoea in children less than 5 years of age worldwide [3]. They are responsible for 453,000 deaths worldwide each year, which in most cases (85%) occur in developing countries [3]. The virus particle is composed of six viral proteins (VPs) called VP1, VP2, VP3, VP4, VP6, and VP7. Among these, the glycoprotein VP7 is located on the outer surface of the virus determining the specific G-type of the strain and plays a role in the development of immunity to infection [4].

We have previously described the presence, in active celiac disease (CD), of a subset of antitransglutaminase IgA antibodies that recognizes the viral protein VP-7 and is able to increase intestinal permeability and induce monocyte activation [5]. We then showed that the antirotavirus VP7 antibodies may be even detected before the CD onset and the detection of antitissue transglutaminase (tTG) and antiendomysium antibodies, showing a predictive role [6]. In addition, we observed that these antibodies were able to induce in human T84 intestinal cell line the modulation of genes involved in biological processes that represents typical features of CD [6]. Taken together, our data seem to provide a link between rotavirus infection and CD.

In this paper, we aim at clarifying some aspects of the pathogenesis of NCGS by a gene-array approach. In particular, we plan to verify the possibility of the involvement of an autoimmune mechanism in the disease. In addition, we also aim at investigating a possible involvement of rotavirus infection in the development of NCGS. For this purpose, we compared the global panel of modulated genes in NCGS to the dataset of human T84 intestinal cells treated with antirotavirus VP7 antibodies, described in our previous work [6], and to a dataset of acute phase of rotavirus infection, downloaded from the GEO (Gene Expression Omnibus) database, searching for transcriptional profiles that may be associated to viral infection.

## 2. Materials and Methods

### 2.1. Patients

We studied a cohort of 16 patients (6 males and 10 females, mean age: 27.3 years) affected by NCGS, attending the Unit of Autoimmune Diseases and the Immunology Unit and Child Neuropsychiatry Unit at the University Hospital of Verona, Italy.

All the enrolled subjects were recruited after informed consent. Main symptoms were headache, dermatitis, chronic urticaria, muscle and joint pain, bloating, abdominal pain, diarrhoea, alternating bowel movements, and fatigue in a variable combination.

Diagnosis of NCGS was established when all the following criteria were met: (1) exclusion of wheat allergy by clinical history and determination of specific IgE; (2) exclusion of celiac disease by absence of celiac-specific antibodies tissue transglutaminase (tTG), endomysium (EMA), and/or deamidated gliadin peptides (DGP); (3) duodenal biopsy with a histological damage grade 0 to 1, according to Marsh's classification; (4) significant improvement of symptoms on strict gluten-free diet and relapse of symptoms after gluten reintroduction.

### 2.2. Detection of Anti-VP7 Peptide Antibodies

The ELISA test for antibody binding to the synthetic peptides has been carried out as already described elsewhere with minor modifications [7]. The synthetic peptides were used at a concentration of 20 *μ*/mL in PBS to coat polystyrene plates (Immulon 2HB, Thermo). For the detection of antirotavirus VP7 peptide IgA antibodies, only the sera whose OD readings were higher than the mean plus three standard deviations of each serum dilution of the control group were considered positive. OD values higher than 0.140 were considered positive.

### 2.3. Gene Array

Peripheral blood cells were collected for analysis of gene expression profiles on a gluten-containing diet. PAXgene Blood RNA tubes (PreAnalytiX, Hombrechtikon, Switzerland) were used for blood collection and total RNA was extracted according to the protocol supplied by the manufacturer. Preparation of cRNA hybridization and scanning of arrays for each samples were performed following the manufacturer instructions (Affymetrix, Santa Clara, CA, USA) by Cogentech Affymetrix microarray unit (Campus IFOM IEO, Milan, Italy) using the Human Genome U133A 2.0 GeneChip (Affymetrix). The gene expression profiles were analysed using the GeneSpring software version 12.1 (Agilent Technologies, Santa Clara, CA, USA) that calculated a robust multiarray average of background-adjusted, normalized, and log-transformed intensity values applying the robust multiarray average algorithm (RMA). The normalized data were transformed to the log_2_ scale. The unpaired *t*-test was performed to determine which genes were modulated at a significant level (*p* ≤ 0.01), and *p* values were corrected for multiple testing by using Bonferroni correction. Finally, statistically significant genes were chosen for final consideration when their expression was at least 1.5-fold different in the test sample versus control sample. Genes that passed both the *p* value and the FC restriction were submitted to functional and pathway enrichment analysis according to the Gene Ontology (GO) annotations employing the Panther expression analysis tools (http://pantherdb.org/).

### 2.4. Protein-Protein Interaction (PPI) Network Construction and Network Modular Analysis

All the possible interactions among the protein products of DEGs were analysed with Search Tool for the Retrieval of Interacting Genes (STRING version 1.0; http://string-db.org/) a web-based database that includes experimental as well as predicted interaction information and covers more than 1100 sequenced organisms. Only protein-protein interaction (PPI) pairs that were confirmed by experimental studies were selected, and a score of ≥0.7 for each PPI pair was used to build a PPI network.

Cytoscape software [8] was used to define the topology of the built network, and the Molecular Complex Detection (MCODE) [9] was used to find densely connected region (modules) of the network that could be involved in the modulation of biological processes that are relevant for the disease pathogenesis. To find locally dense regions of a graph, MCODE applies a vertex-weighting scheme based on a clustering coefficient that is a measure of the degree to which nodes in a graph tend to cluster together.

The following settings in MCODE were used: degree cutoff = 2, K-core = 3, and max. depth = 100. Functional enrichment for a given module was assessed quantitatively using the Panther tool.

### 2.5. Analysis of the Association between DEGs and Human Diseases

We used the software Ingenuity Pathway Analysis (IPA, Ingenuity Systems) to evaluate diseases and disorders that could be statistically significantly associated to gene modulation observed in NCGS samples. The statistical significance of gene-disease associations was calculated in IPA by the Fisher's exact test (*p* ≤ 0.0001).

### 2.6. Detection of Soluble Mediators in GS Sera

Serum levels of sCTLA-4, s PD-1, and sgp130/IL6ST were detected before and after gluten-free diet using commercially available ELISA kits according to the manufacturer's instructions. ELISA kits were purchased from Bender MedSystems (Milano, Italy) (sCTLA-4), from R&D Systems (Minneapolis, United States) (sgp130), and from EMELCA Bioscience (Clinge, Netherlands) (sPD-1).

### 2.7. FACS Analysis

Cells collected from patients and normal controls were cultured at a concentration of 1^∗^10^6^ cells/mL in 2 mL tubes containing 1 mL of RPMI 1640 + FCS 10% (Lonza, Basel, CH). Cells were stimulated overnight with Dynabeads Human T-Activator CD3/CD28 (Life Technologies, Carlsbad, CA, USA). The detection of IL-17 production was analysed using the IL-17 Secretion Assay (Miltenyi Biotec, Bergisch Gladbach, D) following the manufacturer's instruction. Briefly, cells were washed with 2 mL of cold buffer at 300 ×g for 5 minutes at 4°C, and the pellet was resuspended in 90 *μ*L of cold medium. Cells were then incubated with 10 *μ*L of IL-17 Catch Reagent for 5 minutes in ice and cultured in 1 mL of warm medium at 37°C for 45 minutes under slow continuous rotation. Cells were then washed with cold buffer and resuspended in 75 *μ*L of cold buffer; 10 *μ*L of IL-17 Detection Antibody APC, 10 *μ*L of anti-CD3 PerCP (Becton Dickinson, Franklin Lakes, NJ, USA), and 5 *μ*L of anti-CD4 APC-H7 (Becton Dickinson) monoclonal antibodies were added. Incubation was carried out in ice for 10 minutes. Finally, cells were washed and resuspended in an appropriate volume of PBS and acquired on a FACSCanto II cytometer (Becton Dickinson). Analysis was performed with FlowJo 9.3.3 software (Tree Star, Ashland, OR, USA).

### 2.8. Statistical Analysis

Data obtained from the analysis of the soluble mediators CTLA-4, gp130, and PD-1 and from the detection of antigliadin antibodies were submitted to statistical testing using the Wilcoxon nonparametric statistical hypothesis test for paired samples.

Data obtained from the ELISA test for the detection of antirotavirus VP7 peptide antibodies were submitted to statistical testing using the Mann–Whitney nonparametric test. Statistical analysis was performed using GraphPad Prism Software version 5.00 (GraphPad Software, La Jolla, California, USA, http://www.graphpad.com).

## 3. Results and Discussion

Many aspects of NCGS are still unknown; in particular, it is still not clear whether the disease is permanent or transient or whether the disease has features of autoimmunity. The pathogenesis of NCGS is also unclear and data obtained so far suggest a prevalent activation of innate immune responses [2].

We aimed at clarifying some aspects of NCGS pathogenesis using a gene array approach which we successfully used in the study of many immune-mediated diseases [6, 10–12].

In order to identify specific gene signatures typically associated with NCGS, we compared the gene expression profiles of 8 PBC samples obtained from individual NCGS patients with 10 PBC samples obtained from healthy age- and sex-matched donors. We observed that the disease has a profound impact on gene expression profiles since a large number of differentially expressed genes (DEGs) (1293, represented by 1521 modulated probe sets) complied with the Bonferroni-corrected *p* value criterion (*p* ≤ 0.01) and the fold change criterion (FC ≥ 1.5) showing robust and statistically significant variation between healthy controls and NCGS samples. In particular, 695 and 598 genes resulted to be up- and downregulated, respectively (Additional Table 1).

DEGs were submitted to functional enrichment analysis according to terms of the Gene Ontology (GO) biological processes (BP) and canonical pathways. The most enriched biological process was “immune system” followed by “intracellular signal transduction” (Table 1). In addition, several enriched terms were related to the immune response gene category, including “leukocyte differentiation,” “leukocyte activation involved in immune response,” “T cell differentiation,” “neutrophil degranulation,” “adaptive immune response,” and “defense response.” Interestingly, we observed an enrichment in “cellular response to organic substance,” “cellular response to endogenous stimulus,” and “viral process.” The BP named “viral process” is defined by the Gene Ontology Consortium as a “multi-organism process in which a virus is a participant and the other participant is the host.” This term is related to the infection of a host cell, the replication of the viral genome, the viral transcription, and the assembly of progeny virus particles.

Pathway enrichment analysis showed that the most enriched signaling pathways were “inflammation mediated by chemokine and cytokine,” “apoptosis,” and “angiogenesis,” followed by “T cell activation” and “B cell activation” (Table 1). Other enriched pathways were: “integrin signaling,” “EGF receptor signaling,” “Toll-like receptor signaling,” “PI3 kinase,” “interleukin signaling,” and JAK/STAT signaling. Since the majority of the top-enriched functional classes and pathways were related to the immune system, we selected, within the entire data set, all modulated genes associated to the “Immune response” GO term to better characterize the immunological processes that are involved in NCGS pathogenesis. Although both innate and adaptive immunity play a crucial role in the development of CD, NCGS has been mainly associated with activation of the innate immune response [2].

It is therefore surprising to notice that both transcripts involved in the innate immune response as well as genes of the adaptive immune response were well represented in our dataset (Table 2).

In this regard, 14 genes involved in NK activity were modulated in NCGS samples (i.e., LILRA1, LILRA2, CLEC2D, and KLRC4). Moreover, several genes involved in macrophage activation were modulated in NCGS including TNFRSF10B, the ligand of the death receptors TRAIL that play important roles in set up both innate and adaptive immune responses against pathogens [13], and the scavenger receptors MRC1/CD206 [14] and MARCO, a member of the class A scavenger receptor family strongly upregulated in M*Φ* by various microbial stimuli in a TLR-dependent manner [15].

Noteworthy, 38 genes prevalently related to B cell activity (i.e., IL2RG, IL6R, KLF12, and CD27) were also modulated, indicating an important role for this cell subset in NCGS, 20 genes involved in T cell activation were upregulated in NCGS samples (i.e., CD28, CD3E, CD3G, and CTLA-4). Remarkably, Th17-lymphocyte-related genes and transcripts that can modulate Th17 cell development and functions were overexpressed including IL4R, IL2RG, IL6ST, IL1B, IL7R, STAT6, STAT5B, SOCS3, and CXCL2.

DEGs indicate therefore active involvement of both arms of the adaptive immune response (i.e., T and B cells response) and a prevalent upregulation of several Th17-related genes in the T cell response category. It is well known that Th17 cells play an important role in autoimmunity and have been implicated in the pathogenesis of psoriasis and in the amplification of inflammation in rheumatoid synovitis and in lupus nephritis [16–18].

In the NCGS dataset, 6 type I interferon inducible genes (IFIG) were upregulated (IFNA17, IRF5, IRF3, STAT2, STAT1, and LY9), thus indicating the presence of an IFN type I signature, typically associated with autoimmune disease such as systemic lupus erythematosus (SLE), rheumatoid arthritis (RA), Crohn's disease, and Sjogren syndrome [19–25].

In this respect, it is well known that Th17 cells and related cytokines are crucial in promoting autoimmunity, in particular, when they act in synergy with type I IFN-driven inflammation. In the presence of IFN type I signature, CCR6^+^ memory T-helper cells producing IL-17A, IL-17F, IL-21, and/or IL-22 are increased in SLE, [26] indicating that, in the pathogenesis of systemic autoimmune diseases, IFN type I signature coacts with Th17 cells and related cytokines.

**Figure 1:**
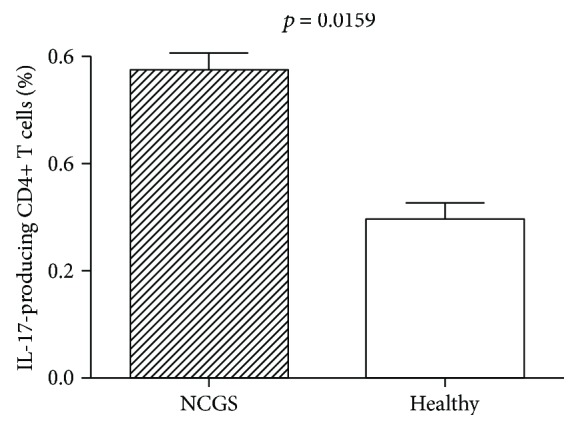
Flow cytometric analysis of CD4+T cells releasing IL-17 in patients with NCGS. Panel displays the mean percentage of CD4+T cells releasing IL-17 of 10 healthy donors and 8 NCGS patients. PBMCs were stimulated overnight with anti-CD3/-CD28-coated beads. *p* value calculated with the Mann–Whitney nonparametric statistical test was 0.0159.

In order to further confirm our gene expression data on overexpression of IFIG and Th17 pathways, we analysed the presence of IL-17-producing CD4+ T cells and found a significantly (*p* = 0.0159) increased percentage of these cells in PBMC of patients with NCGS compared with normal subjects (Figure 1).

**Figure 2:**
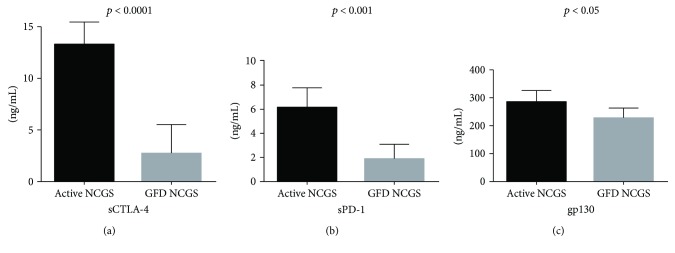
Serum levels of selected soluble mediators in NCGS patients and in normal subject sera. The histograms represent the mean of the results obtained in 20 healthy donors and in 16 NCGS patients. *p* values calculated with the Wilcoxon nonparametric statistical test for paired samples were: *p* < 0.0001 for sCTLA-4, *p* < 0.001 for sPD-1, and *p* < 0.05 for sgp130.

The analysis of genes modulated in gluten sensitivity was paralleled by the detection of some of the corresponding soluble mediators in the sera of NCGS patients. We analysed selected molecules that are widely recognized to be associated to an autoimmune response, including sCTLA-4, sPD-1, and sgp130/IL6ST.  Figure 2 shows the concentration of these molecules in the sera of NCGS patients before and after gluten-free diet. The serum levels of all the molecules tested were significantly higher in NCGS before GFD than after GFD.

**Figure 3:**
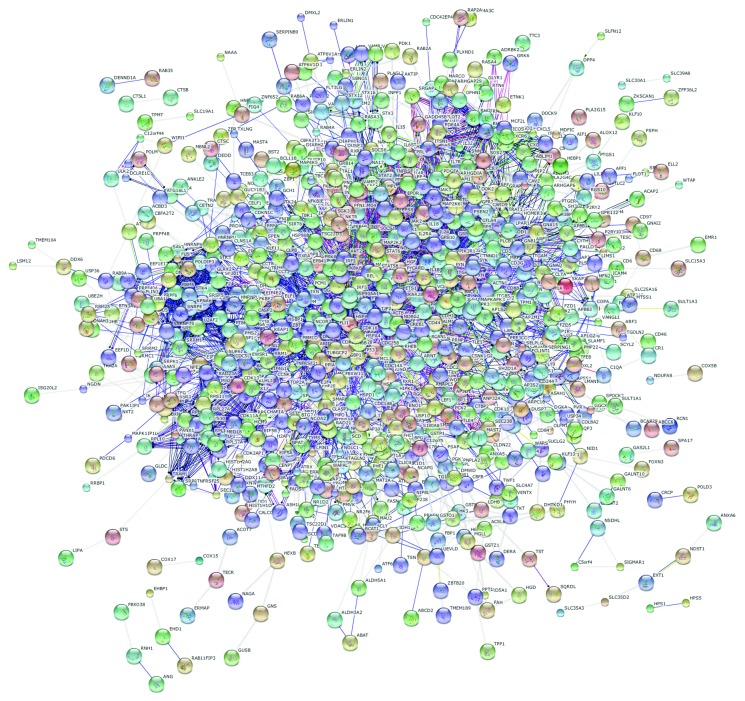
Protein-protein interaction (PPI) network of DEGs in NCGS patients.

**Figure 4:**
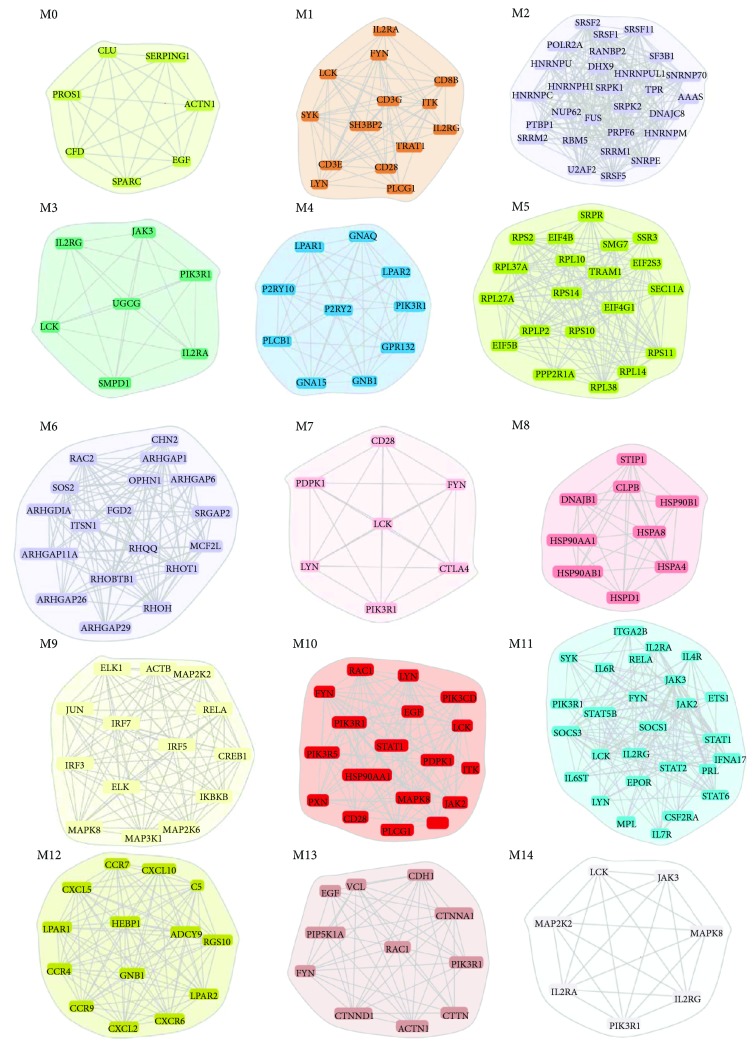
Modules originated from the network analysis of DEGs in NCGS patients.

In order to gain further insights into the molecular mechanisms relevant in NCGS pathogenesis, we constructed a protein-protein interaction (PPI) network starting from all the 1293 DEGs. The resulted PPI network contained 853 nodes and 3512 edges (Figure 3). By performing a modular analysis of the constructed PPI network, we were able to identify clusters of the most densely interconnected nodes (modules) and to extrapolate 15 main modules of genes displaying the highest degree of connection.  Figure 4 shows a graphical representation of such modules, where the nodes represent proteins and the edges indicate their relations.

All modules were submitted to enrichment analysis to find enriched GO biological processes and pathways.

Among the 15 modules in particular, five (module 1, 3, 7, 10, and 14) showed a prevalent enrichment in BP and pathways associated to the activation of T cells. Similarly, “B cell activation” pathways were significantly enriched in modules 1, 9, 10, and 14. Interestingly, in modules 3, 10, and 11, we observed an enrichment in the JAK–STAT signaling pathway, which is highly relevant to human autoimmunity [27] and plays a role in the intestinal mucosal immune homeostasis as well as in intestinal epithelial repair and regeneration [28]. We also observed that module 11 contained several genes involved in Th-17 cell functions (i.e., IL2RG, IL4R, IL6ST, IL7R, SOCS3, STAT5B, and STAT6) and several IFIG, including IFNA17, STAT1, and STAT2. Other IFIG genes were ascribed to module 9 which also shows an enrichment in BPs associated to type I interferon signaling, including positive regulation of type I interferon production, positive regulation of interferon-beta production, and type I interferon biosynthetic process (Table 3).

Loss of the intestinal barrier integrity is a typical feature of CD and represents an important mechanism of autoimmunization through the passage of antigens across the intestinal epithelium [29]. However, Sapone et al. [29] have shown that NCGS patients have normal intestinal permeability when compared to CD patients, as assessed by the lactulose-mannitol test.

Indeed, in module 13, in which the most enriched BP was “adherent junction assembly,” we observed a reduced expression of molecules involved in cell adhesion including CDH1 (epithelial cadherin), CTNNA1, VCL, and CTTN, a molecule expressed on the apical surface of the polarized epithelium. In the same module, we also observed underexpression of Rac1, a critical regulator of intestinal epithelial barrier functions [30] and EGF, known to protect intestinal barrier integrity by stabilizing the microtubule cytoskeleton [31] and upregulation of FYN and PIK3R1, both involved in the signaling pathway by which IFN*γ* increases intestinal permeability [32].

The gene expression data would therefore indicate deregulation of adherent junctions and altered intestinal permeability also in NCGS, which seems to be in contrast with the data of Sapone et al. Nevertheless, it is important to point out that the lactulose-mannitol test may not be sensitive enough to detect mild alterations of the intestinal barrier function in patients with NCGS.

In module 12, the most enriched pathway was “inflammation mediated by chemokine and cytokine signaling”; this pathway was also enriched in modules 9, 10, and 11, which is consistent with inflammatory/autoimmune origin of NCGS.

Moreover, modules 1, 2, 7, and 10 were enriched in BPs related to viral infection including “viral process,” “viral gene expression,” “intracellular transport of virus,” and “regulation of defense response to virus.”

In addition, we observed that modules 10 and 11 showed enrichments in the gamma interferon pathways typically associated to the innate response to viruses [33].

**Figure 5:**
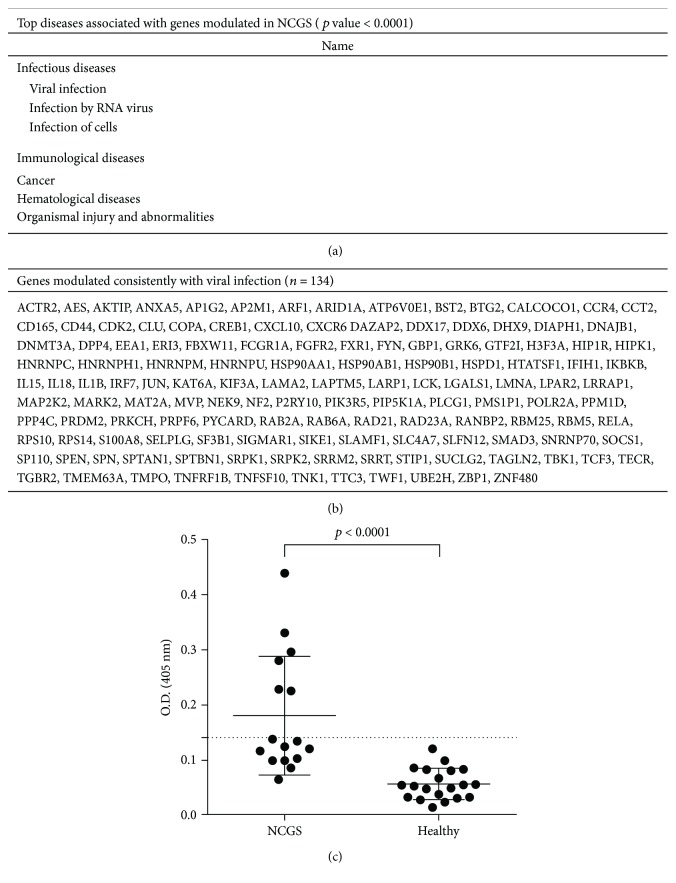
(a) List of diseases which are most likely to be statistically significantly associated and compatible with the transcriptional profile observed in NCGS. (b) DEGs in NCGS showing a modulation consistent with a viral infection process. (c) Detection of antibodies directed against the rotavirus VP7 peptide in the sera of patients with NCGS. Each circle represents a measurement for one patient, and the dashed horizontal line indicates the threshold for positivity (O.D. 0.140). The statistical *p* value was calculated with the Mann–Whitney test (*p* < 0.0001).

Therefore, to further clarify the relationship between viral infections and NCGS, we searched in the IPA software database to find all diseases that are most likely to be statistically significantly associated to the genes modulated in the NCGS dataset. We found that, in the resulting list of most significantly associated diseases, “Infectious diseases” ranked first and, among these, “Viral infection” showed the best statistical *p* value (Figure 5(a)). Moreover, we could find a cluster of 134 DEGs that, in our NCGS dataset, showed a modulation that was consistent with a process of viral infection (Figure 5(b)). Based on these data, we aimed at investigating whether rotavirus, known to be linked to CD, [5, 6, 34] could also play a role in NCGS.

In the second part of our study, we made a comparison between the dataset obtained from our previous analysis of intestinal human T84 cells treated with anti-VP7 antibodies (that we indicate in this paper as “T84 dataset”) and genes modulated in NCGS. We found that 529 genes modulated in NCGS (accounting for the 41% of genes modulated in this dataset) were also modulated in treated T84 cells. Interestingly, several DEGs that were shared by the two datasets are involved in BP that may be related to the pathogenesis of celiac disease, including apoptosis, inflammatory and immune response, cell proliferation, cell differentiation, cell junctions, matrix metalloproteases, receptors and signal transducers, cytoskeleton components, ion transport and exchange, and EGF receptor pathway.  Table 4 shows a selection of genes ascribed to the abovementioned functional classes. As a whole in NCGS dataset, the modulation of genes ascribed to the abovementioned categories indicated an upregulation of apoptotic genes accompanied by a downregulation of genes involved in cell differentiation and an increased transcription of proliferative genes. All these observation are in agreement with what we described on human T84 cells treated with antirotavirus Vp7 peptide antibodies and are related to the typical features of celiac disease. Indeed in CD, an increased apoptosis is the main cause of villous atrophy that is also sustained by a dysregulation of cell differentiation [35]. Moreover, it has been observed that the increase of intestinal cell proliferation leads to crypt hyperplasia seen in celiac disease [35]. Other aspects of CD previously observed in our T84 treated cells, that are paralleled by the gene modulated observed in NCGS, are the upregulation of members of the epidermal growth factor receptor (EGFR) signaling pathway and the concomitant downregulation of cell adhesion molecules beside a deregulation of ion transport. Noteworthy, the activation of EGFR signaling has been already observed in CD [36], and dysfunction of cell adhesion and transport are typical features of epithelial cells from active CD [37].

In this regard, it is worthwhile mentioning that patients with NCGS have normal to mildly inflamed mucosa (Marsh 0-1), while partial or subtotal villous atrophy and crypt hyperplasia are hallmarks of CD. Nevertheless, we cannot exclude that some NCGS patients, especially those positive for HLA-DQ2 and/or DQ8, may switch to classical CD in the course of the follow-up.

Since a large part of DEGs in the NCGS paralleled the modulation of genes seen in human T84 cells treated with antirotavirus Vp7 peptide antibodies, we next aimed at identifying the presence of such antibodies in sera of NCGS patients. We therefore tested in ELISA assay the sera from 16 NCGS patients and 20 healthy subjects for the detection of antirotavirus VP7 peptide antibodies. We found that these antibodies were present in 6 out of 16 (37%) NCGS patients while were not detected in the sera of healthy subjects.  Figure 5(c) shows that the levels of such antibodies are significantly different in the two set of tested samples (*p* < 0.0001). The detection of these antibodies in NCGS patients may be relevant to the pathogenesis of the NCGS given their ability to modulate sets of genes in intestinal epithelial cells as we previously demonstrated [6].

Taken together, the modulation of highly connected genes associated to the viral infection process and the presence of anti-VP7 antibodies in the sera of some NCGS patients may suggest that a link also exists between immune response to rotavirus infection and NCGS.

In this perspective, since anti-VP7 rotavirus antibodies are often present before the onset of CD, preceding the detection of celiac specific autoantibodies, [6] it is tempting to speculate that NCGS patients with CD genetic predisposition (DQ2/DQ8) and presence of anti-VP7 antibodies may develop CD in the course of the follow-up.

Therefore, to further clarify the relationship between rotavirus infection and NCGS, we decided to perform an integrative bioinformatics analysis using the dataset GSE50628 downloaded from GEO (Gene Expression Omnibus) database (http://www.ncbi.nlm.nih.gov/geo/) that included samples of peripheral blood cells from patients affected by acute rotavirus infection (named in the paper “Rotavirus infection dataset”). This dataset was analysed to detect significantly modulated genes (Additional Table 2), and a comprehensive GO analysis was carried out on all datasets including NCGS, Rotavirus infection, and T84 datasets that we analysed in our previous work [6].

We then searched on the four datasets for the presence of genes associated to GO terms containing the words “virus” and/or “viral” and we found in all datasets a great number of such terms to which modulated genes were connected/related.

The searched terms explored a wide range of biological processes associated to viral infection from the entry of virus in the host cell, viral transcription and gene expression, modulation of host physiology by virus to cellular response to virus.

All the GO terms retrieved in the three datasets are listed in Additional Table 3.

Table 5 shows selected genes modulated in the three datasets that are ascribed to the most representative GO terms, including viral transcription, viral gene expression, response to virus, viral genome replication, and viral life cycle.

Moreover, the GO analysis of the abovementioned datasets was complemented by searching for transcripts involved in immune response.

In the “T84 dataset,” we found upregulation for the T cell costimulatory molecule ICOSLG, the transcriptional regulator that is crucial for the development and inhibitory function of regulatory T cells, [38] interleukin-6 that is pivotal for the development of Th-17 cells [39], and FCGR2B that is involved in the phagocytosis of immune complexes and in modulation of antibody production by B cells [40] (Table 6).

In the “Rotavirus infection” dataset, we found upregulation for molecules that are crucial in the immune response including the BLK gene, involved in transmitting signals through surface immunoglobulins, supporting the pro-B to pre-B transition, [41] MR1/MAIT playing a role in the development of the mucosal-associated invariant T cells (MAIT), [42] TNFRSF4 involved in T cell proliferation [43], and HCST/DAP10 playing a role in triggering cytotoxicity against both stressed and infected by virus target cells [44] (Table 7).

Interestingly, in all the datasets, we found the presence of modulated genes involved in the type I interferon signaling, that is central in autoimmunity, and in molecular pathways involved in the immune response to viral infection, including the Toll-like receptors, and the type I and gamma interferon pathways (see Tables 2, 6, and 7).

Taken together, our data seem to indicate that NCGS has features of autoimmunity and that an immune response to rotavirus may play a role in some cases of NCGS.

## 4. Conclusions

NCGS is an emerging new clinical entity lacking specific diagnostic biomarkers which has been reported to occur in 6–10% of the population. Interestingly, up to 50% of these patients carry HLA-DQ2 and/or HLA-DQ8 genes. NCGS patients may complain gastrointestinal (e.g., diarrhoea/constipation, abdominal pain, bloating) and/or extraintestinal symptoms (“foggy mind,” headache, dermatitis, etc.) which recede with GFD. The pathogenesis of NCGS is still unclear and the data, so far obtained, suggest a predominant activation of the innate immune responses.

Our data indicate a concomitant involvement of the adaptive immune system and suggest that NCGS may have an autoimmune origin. This is based both on gene expression data (i.e., TH17-IFNA I signatures) and on the presence of TH17 cells and of serological markers of autoimmunity in NCGS. Our results also indicate a possible involvement of rotavirus infection in the pathogenesis of NCGS, similarly to what we have previously shown in CD.

## Figures and Tables

**Table 1:** Biological processes and pathways that were enriched in the NCGS dataset.

*Biological processes*	*p value* ^∗^
Immune system process	6.3 × 10^−26^
Intracellular signal transduction	4.6 × 10^−16^
Cellular response to organic substance	1.5 × 10^−13^
Cell surface receptor signaling pathway	8.2 × 10^−10^
Leukocyte differentiation	6.3 × 10^−9^
Viral process	7.7 × 10^−9^
Leukocyte activation involved in immune response	8.0 × 10^−8^
Apoptotic process	2.2 × 10^−6^
Cellular response to endogenous stimulus	3.0 × 10^−6^
T cell differentiation	5.6 × 10^−5^
Neutrophil degranulation	5.6 × 10^−5^
Adaptive immune response	6.5 × 10^−5^
Defense response	6.8 × 10^−5^
*Pathways*	*p value* ^∗^
Inflammation mediated by chemokine and cytokine signaling pathway	2.1 × 10^−7^
Apoptosis signaling pathway	1.6 × 10^−4^
Angiogenesis	4.1 × 10^−4^
T cell activation	5.3 × 10^−4^
B cell activation	5.7 × 10^−4^
Integrin signaling pathway	7.8 × 10^−4^
EGF receptor signaling pathway	4.0 × 10^−3^
Toll like receptor signaling pathway	4.6 × 10^−3^
PI3 kinase pathway	7.6 × 10^−3^
Interleukin signaling pathway	8.1 × 10^−3^
JAK/STAT signaling pathway	1.6 × 10^−2^

^∗^Bonferroni corrected.

**Table 2:** Genes modulated in NCGS patients that are involved in immune response and molecular signalings.

Probe set ID	*p* value	Gene symbol	Gene title	FC	Representative public ID
*T cell activation*					
203809_s_at	<0.001	AKT2	v-akt murine thymoma viral oncogene homolog 2	2.36	NM_001626
211861_x_at	<0.001	CD28	CD28 molecule	2.75	AF222343
205456_at	<0.001	CD3E	CD3e molecule, epsilon (CD3-TCR complex)	2.67	NM_000733
206804_at	<0.001	CD3G	CD3g molecule, gamma (CD3-TCR complex)	2.13	NM_000073
211027_s_at	<0.001	IKBKB	Inhibitor of kappa light polyp. gene enhancer in B cells, kinase *β*	2.69	NM_001190720
213281_at	0.007	JUN	jun proto-oncogene	3.07	NM_002228.3
204890_s_at	<0.001	LCK	Lymphocyte-specific protein tyrosine kinase	2.05	U07236
213490_s_at	<0.001	MAP2K2	Mitogen-activated protein kinase kinase 2	1.83	NM_030662
214786_at	0.013	MAP3K1	Mitogen-activated protein kinase kinase kinase 1	1.52	NM_005921
210671_x_at	<0.001	MAPK8	Mitogen-activated protein kinase 8	2.33	NM_001278548
211230_s_at	<0.001	PIK3CD	Phosphatidylinositol-4,5-bisphosphate 3-kinase, catalytic sub. Δ	1.88	U57843
212249_at	0.002	PIK3R1	Phosphoinositide-3-kinase, regulatory subunit 1 (alpha)	2.76	NM_181523
216551_x_at	0.001	PLCG1	Phospholipase C, gamma 1	1.53	NM_002660
208640_at	<0.001	RAC1	Rho family, small GTP-binding protein Rac1	−1.92	NM_006908
207419_s_at	<0.001	RAC2	Rho family, small GTP-binding protein Rac2	2.31	NM_002872
217576_x_at	0.002	SOS2	Son of sevenless homolog 2	1.90	NM_006939
216042_at	<0.001	TNFRSF25	Tumor necrosis factor receptor superfamily, member 25	2.40	NM_148965
221331_x_at	<0.001	CTLA4	Cytotoxic T-lymphocyte-associated protein 4	2.26	NM_005214
206569_at	<0.001	IL24	Interleukin 24	2.84	NM_006850
203828_s_at	0.003	IL32	Interleukin 32	2.10	NM_004221
*B cell mediated immune response*					
211027_s_at	<0.001	IKBKB	Inhibitor of kappa light polyp. gene enhancer in B cells, kinase *β*	2.69	NM_001190720
213281_at	0.007	JUN	jun proto-oncogene	3.07	NM_002228.3
202626_s_at	0.004	LYN	v-yes-1 Yamaguchi sarcoma viral related oncogene homolog	−2.22	AI356412
213490_s_at	<0.001	MAP2K2	Mitogen-activated protein kinase kinase 2	1.83	NM_030662
210671_x_at	<0.001	MAPK8	Mitogen-activated protein kinase 8	2.33	NM_001278548
211230_s_at	<0.001	PIK3CD	Phosphatidylinositol-4,5-bisphosphate 3-kinase, catalytic sub. Δ	1.88	U57843
32540_at	<0.001	PPP3CC	Protein phosphatase 3, catalytic subunit, gamma isozyme	2.00	NM_001243975
208640_at	<0.001	RAC1	Rho family, small GTP-binding protein Rac1	−1.92	NM_006908
207419_s_at	<0.001	RAC2	Family, small GTP-binding protein Rac2	2.31	NM_002872
217576_x_at	0.002	SOS2	Son of sevenless homolog 2	1.90	NM_006939
207540_s_at	0.008	SYK	Spleen tyrosine kinase	−1.92	NM_003177
207224_s_at	0.003	SIGLEC7	Sialic acid-binding Ig-like lectin 7	−2.21	NM_016543
206150_at	0.004	CD27	CD27 molecule	1.96	NM_001242
214447_at	0.005	ETS1	v-ets erythroblastosis virus E26 oncogene homolog 1	2.25	NM_005238
201328_at	0.012	ETS2	v-ets erythroblastosis virus E26 oncogene homolog 2	−1.83	NM_005239
212420_at	0.001	ELF1	E74-like factor 1 (ets domain transcription factor)	1.94	KJ896761
211825_s_at	<0.001	FLI1	Friend leukemia virus integration 1	2.28	AF327066
215967_s_at	<0.001	LY9	Lymphocyte antigen 9	2.08	NM_002348
210690_at	0.001	KLRC4	Killer cell lectin-like receptor subfamily C, member 4	2.23	U96845
204116_at	<0.001	IL2RG	Interleukin 2 receptor, gamma	1.84	NM_000206
217489_s_at	<0.001	IL6R	Interleukin 6 receptor	1.79	S72848
204863_s_at	<0.001	IL6ST	Interleukin 6 signal transducer (gp130, oncostatin M receptor)	4.52	NM_002184
206966_s_at	<0.001	KLF12	Kruppel-like factor 12	1.83	AH010423
219878_s_at	<0.001	KLF13	Kruppel-like factor 13	1.89	NM_015995
219386_s_at	<0.001	SLAMF8	SLAM family member 8	−2.27	NM_020125
210405_x_at	0.003	TNFRSF10B	Tumor necrosis factor receptor superfamily, member 10b	1.50	NM_003842
219386_s_at	<0.001	SLAMF8	SLAM family member 8	−2.27	NM_020125
210405_x_at	0.003	TNFRSF10B	Tumor necrosis factor receptor superfamily, member 10b	1.50	NM_003842
203508_at	0.005	TNFRSF1B	Tumor necrosis factor receptor superfamily, member 1B	−2.06	NM_001066
216042_at	<0.001	TNFRSF25	Tumor necrosis factor receptor superfamily, member 25	2.40	NM_148965
218856_at	0.008	TNFRSF21	Tumor necrosis factor receptor superfamily, member 21	−1.68	NM_014452
206181_at	0.003	SLAMF1	Signaling lymphocytic activation molecule family member 1	1.65	NM_003037
210796_x_at	<0.001	SIGLEC6	Sialic acid-binding Ig-like lectin 6	1.58	D86359
211192_s_at	<0.001	CD84	CD84 molecule	2.55	AF054818
220132_s_at	<0.001	CLEC2D	C-type lectin domain family 2, member D	3.18	NM_013269
204773_at	0.005	IL11RA	Interleukin 11 receptor, alpha	1.56	AY532110
210850_s_at	<0.001	ELK1	ELK1, member of ETS oncogene family	1.60	AF000672
209894_at	0.002	LEPR	Leptin receptor	−2.10	U50748
203005_at	0.006	LTBR	Lymphotoxin beta receptor (TNFR superfamily, member 3)	−1.92	NM_002342
*NK cell activation*					
220132_s_at	<0.001	CLEC2D	C-type lectin domain family 2, member D	3.18	NM_013269
203233_at	0.014	IL4R	Interleukin 4 receptor	1.50	NM_000418
210152_at	0.007	LILRB4	Leukocyte immunoglobulin-like receptor, subfamily B, member 4	−1.83	NM_001278426
210784_x_at	0.012	LILRA6	Leukocyte immunoglobulin-like receptor, subfamily A, member 6	−1.74	NM_024318
211405_x_at	0.003	IFNA17	Interferon, alpha 17	1.59	NM_021268
210660_at	0.008	LILRA1	Leukocyte immunoglobulin-like receptor, subfamily A, member 1	−2.81	NM_001278319
207857_at	0.016	LILRA2	Leukocyte immunoglobulin-like receptor, subfamily A, member 2	−2.35	NM_006866
210690_at	0.001	KLRC4	Killer cell lectin-like receptor subfamily C, member 4	2.23	U96845
206881_s_at	0.013	LILRA3	Leukocyte immunoglobulin-like receptor, subfamily A, member 3	−2.96	NM_006865
210313_at	0.003	LILRA4	Leukocyte immunoglobulin-like receptor, subfamily A, member 4	−1.83	NM_012276
215838_at	0.012	LILRA5	Leukocyte immunoglobulin-like receptor, subfamily A, member 5	−3.15	NM_181985
210146_x_at	0.004	LILRB2	Leukocyte immunoglobulin-like receptor, subfamily B, member 2	−3.41	AF004231
208982_at	0.008	PECAM1	Platelet/endothelial cell adhesion molecule 1	−1.73	M37780
203828_s_at	0.003	IL32	Interleukin 32	2.10	NM_004221
*Macrophage activation*					
210405_x_at	0.003	TNFRSF10B	Tumor necrosis factor receptor superfamily, member 10b	1.50	NM_003842
221900_at	0.003	COL8A2	Collagen, type VIII, alpha 2	−1.62	NM_005202
205819_at	<0.001	MARCO	Macrophage receptor with collagenous structure	−3.01	NM_006770
208602_x_at	<0.001	CD6	CD6 molecule	3.67	NM_006725
207540_s_at	0.008	SYK	Spleen tyrosine kinase	−1.92	NM_003177
203508_at	0.005	TNFRSF1B	Tumor necrosis factor receptor superfamily, member 1B	−2.06	NM_001066
204438_at	0.012	MRC1	Mannose receptor, C type 1	−2.04	NM_002438
202269_x_at	0.006	GBP1	Guanylate binding protein 1, interferon-inducible	−1.77	NM_002053
208982_at	0.008	PECAM1	Platelet/endothelial cell adhesion molecule 1	−1.73	M37780
*Complement activation*					
205500_at	0.006	C5	Complement component 5	−1.60	NM_001735
206244_at	0.003	CR1	Complement component (3b/4b) receptor 1 (Knops blood group)	−1.79	NM_000573
*Response to gamma interferon*					
205831_at	0.001	CD2	CD2 molecule	2.00	NM_001767
205468_s_at	0.015	IRF5	Interferon regulatory factor 5	1.52	NM_032643
219386_s_at	<0.001	SLAMF8	SLAM family member 8	−2.27	NM_020125
211192_s_at	<0.001	CD84	CD84 molecule	2.55	AF054818
202269_x_at	0.006	GBP1	Guanylate-binding protein 1, interferon-inducible	−1.77	NM_002053
202621_at	<0.001	IRF3	Interferon regulatory factor 3	1.67	NM_001571
33148_at	<0.001	ZFR	Zinc finger RNA binding protein	2.19	NM_016107
206181_at	0.003	SLAMF1	Signaling lymphocytic activation molecule family member 1	1.65	NM_003037
215967_s_at	<0.001	LY9	Lymphocyte antigen 9	2.08	NM_002348
201461_s_at	0.011	MAPKAPK2	Mitogen-activated protein kinase-activated protein kinase 2	1.85	NM_004759
216450_x_at	<0.001	HSP90B1	Heat shock protein 90 kDa beta (Grp94), member 1	3.76	AK025862
214370_at	<0.001	S100A8	S100 calcium-binding protein A8	3.65	AW238654
*Antigen processing and presentation*					
206050_s_at	0.010	RNH1	Ribonuclease/angiogenin inhibitor 1	−1.53	NM_002939
204770_at	<0.001	TAP2	Transporter 2, ATP-binding cassette, sub-family B (MDR/TAP)	1.86	NM_000544
*TH17 related genes*					
203233_at	0.014	IL4R	Interleukin 4 receptor	1.50	NM_000418
204116_at	<0.001	IL2RG	Interleukin 2 receptor, gamma	1.84	NM_000206
204863_s_at	<0.001	IL6ST	Interleukin 6 signal transducer (gp130, oncostatin M receptor)	4.52	NM_002184
205067_at	0.015	IL1B	Interleukin 1, beta	1.52	NM_000576
205798_at	0.011	IL7R	Interleukin 7 receptor	1.55	NM_002185
201332_s_at	0.013	STAT6	Signal transducer and activator of transcription 6	1.54	AH006951
205026_at	<0.001	STAT5B	Signal transducer and activator of transcription 5B	1.60	NM_012448
206360_s_at	<0.001	SOCS3	Suppressor of cytokine signaling 3	1.83	NM_003955
209774_x_at	0.015	CXCL2	Chemokine (C-X-C motif) ligand 2	1.53	M57731
*Type I interferon signaling*					
211405_x_at	0.003	IFNA17	Interferon, alpha 17	1.59	NM_021268
205468_s_at	0.015	IRF5	Interferon regulatory factor 5	1.52	NM_032643
202621_at	<0.001	IRF3	Interferon regulatory factor 3	1.67	NM_001571
217199_s_at	<0.001	STAT2	Signal transducer and activator of transcription 2, 113 kDa	1.59	S81491
M97935_5_at	<0.001	STAT1	Signal transducer and activator of transcription 1, 91 kDa	2.73	NM_007315
210370_s_at	<0.001	LY9	Lymphocyte antigen 9	2.05	NM_002348

**Table 3:** Biological processes and pathways enriched in the 15 modules.

Biological processes	*p* value	Pathways	*p* value
*M0*			
Exocytosis	<0.001	None	
Secretion by cell	<0.001		
Secretion	<0.001		
Vesicle-mediated transport	0.0018		
Single-organism transport	0.0220		
Single-organism localization	0.0308		
*M1*			
T cell receptor signaling pathway	<0.001	T cell activation	<0.001
Transmembrane receptor protein tyrosine kinase signaling pathway	<0.001	B cell activation	0.0012
T cell costimulation	<0.001	Cadherin signaling pathway	0.0056
Viral process	<0.001	Integrin signaling pathway	0.0081
Fc-gamma receptor signaling pathway involved in phagocytosis	<0.001		
Peptidyl-tyrosine modification	0.0016		
Adaptive immune response	0.0017		
Positive regulation of antigen receptor-mediated signaling pathway	0.0029		
Positive regulation of alpha-beta T cell proliferation	0.0038		
Phosphatidylinositol phosphorylation	0.0060		
Phosphatidylinositol-mediated signaling	0.0162		
Positive regulation of calcium-mediated signaling	0.0192		
T cell selection	0.0244		
Leukocyte migration	0.0303		
Interleukin-2-mediated signaling pathway	0.0324		
MAPK cascade	0.0371		
Positive regulation of immune effector process	0.0466		
Positive regulation of defense response	0.0485		
*M2*			
mRNA export from nucleus	<0.001	None	
Spliceosomal complex assembly	<0.001		
Termination of RNA polymerase II transcription	<0.001		
Regulation of mRNA splicing, via spliceosome	<0.001		
Positive regulation of RNA splicing	<0.001		
mRNA 3′-end processing	<0.001		
Regulation of gene silencing by miRNA	<0.001		
tRNA export from nucleus	0.0010		
Viral gene expression	0.0054		
Intracellular transport of virus	0.0078		
Protein sumoylation	0.0294		
Regulation of cellular response to heat	0.0310		
Fibroblast growth factor receptor signaling pathway	0.0414		
*M3*			
Positive regulation of T cell activation	0.0035	T cell activation	<0.001
Interleukin-2-mediated signaling pathway	0.0065	Interleukin signaling pathway	<0.001
Interleukin-4-mediated signaling pathway	0.0065	PDGF signaling pathway	0.0010
Protein phosphorylation	0.0349	Integrin signaling pathway	0.0017
		JAK/STAT signaling pathway	0.0057
		Hypoxia response via HIF activation	0.0110
		Insulin/IGF pathway-protein kinase B signaling cascade	0.0136
		p53 pathway feedback loops 2	0.0176
		PI3 kinase pathway	0.0182
		VEGF signaling pathway	0.0238
		Endothelin signaling pathway	0.0284
		p53 pathway	0.0290
*M4*			
Pospholipase C-activating G-protein-coupled receptor signaling pathway	<0.001	Heterotrimeric G-protein signal. pathway-Gq *α* and Go *α* med. pathway	<0.001
G-protein coupled acetylcholine receptor signaling pathway	<0.001		
Activation of phospholipase C activity	<0.001	PI3 kinase pathway	<0.001
Positive regulation of cytosolic calcium ion concentration	<0.001	Endothelin signaling pathway	0.0013
Adenylate cyclase-modulating G-protein-coupled receptor signaling pathway	0.0048	Wnt signaling pathway	0.0015
*M5*			
Translational initiation	<0.001	None	
Nuclear-transcribed mRNA catabolic process, nonsense mediated decay	<0.001		
SRP-dependent cotranslational protein targeting to membrane	<0.001		
rRNA processing	<0.001		
Ribosomal small subunit assembly	0.0083		
*M6*			
Regulation of small GTPase-mediated signal transduction	<0.001	None	
Positive regulation of GTPase activity	<0.001		
Small GTPase-mediated signal transduction	<0.001		
Actin cytoskeleton organization	0.0108		
*M7*			
T cell costimulation	<0.001	T cell activation	<0.001
Phosphatidylinositol-mediated signaling	<0.001	Integrin signaling pathway	0.0041
T cell receptor signaling pathway	<0.001		
Phosphatidylinositol phosphorylation	<0.001		
Transmembrane receptor protein tyrosine kinase signaling pathway	0.0016		
Peptidyl-tyrosine autophosphorylation	0.0033		
Viral process	0.0035		
Fc receptor signaling pathway	0.0050		
Regulation of apoptotic process	0.0055		
Leukocyte differentiation	0.0122		
Leukocyte migration	0.0232		
Lymphocyte activation	0.0237		
B cell receptor signaling pathway	0.0256		
Positive regulation of defense response	0.0340		
*M8*			
Response to unfolded protein	<0.001	None	
Response to topologically incorrect protein	<0.001		
Chaperone-mediated protein complex assembly	<0.001		
Protein folding	<0.001		
Protein transmembrane transport	<0.001		
Response to stress	<0.001		
*M9*			
Activation of innate immune response	<0.001	Toll-like receptor signaling pathway	<0.001
Positive regulation of innate immune response	<0.001	Ras pathway	<0.001
Toll-like receptor signaling pathway	<0.001	Apoptosis signaling pathway	<0.001
Fc-epsilon receptor signaling pathway	0.0020	T cell activation	<0.001
MAPK cascade	0.0026	p38 MAPK pathway	<0.001
Positive regulation of type I interferon production	0.0029	Oxidative stress response	<0.001
Positive regulation of cytokine production	0.0035	Angiogenesis	<0.001
TRIF-dependent toll-like receptor signaling pathway	0.0136	B cell activation	<0.001
Positive regulation of interferon-beta production	0.0202	FGF signaling pathway	<0.001
Response to lipopolysaccharide	0.0268	EGF receptor signaling pathway	<0.001
Type I interferon biosynthetic process	0.0419	Integrin signaling pathway	0.0024
		Inflammation mediated by chemokine and cytokine signaling pathway	0.0079
		
		Interleukin signaling pathway	0.0104
*M10*			
T cell receptor signaling pathway	<0.001	T cell activation	<0.001
T cell costimulation	<0.001	EGF receptor signaling pathway	<0.001
Fc-epsilon receptor signaling pathway	<0.001	Integrin signaling pathway	<0.001
phosphatidylinositol phosphorylation	<0.001	p53 pathway feedback loops 2	<0.001
Peptidyl-tyrosine autophosphorylation	<0.001	VEGF signaling pathway	<0.001
Fc-gamma receptor signaling pathway involved in phagocytosis	<0.001	B cell activation	<0.001
Leukocyte migration	<0.001	Ras pathway	<0.001
Growth hormone receptor signaling pathway	<0.001	Angiogenesis	<0.001
Regulation of defense response to virus	<0.001	Insulin/IGF pathway-protein kinase B signaling cascade	<0.001
Innate immune response	<0.001		
Positive regulation of MAP kinase activity	<0.001	Inflammation mediated by chemokine and cytokine signaling pathway	<0.001
T cell differentiation	<0.001		
Regulation of apoptotic process	<0.001	PI3 kinase pathway	<0.001
JAK–STAT cascade	0.0011	p53 pathway	<0.001
Positive regulation of immune effector process	0.0031	Interferon-gamma signaling pathway	<0.001
MAPK cascade	0.0056	FGF signaling pathway	<0.001
Adaptive immune response	0.0088	Endothelin signaling pathway	0.0101
B cell receptor signaling pathway	0.0121	JAK/STAT signaling pathway	0.0176
Phosphatidylinositol 3-kinase signaling	0.0214		
Stimulatory C-type lectin receptor signaling pathway	0.0363		
Innate immune response activ. cell surface receptor signal. pathway	0.0387		
*M11*			
Cellular response to cytokine stimulus	<0.001	JAK/STAT signaling pathway	<0.001
JAK–STAT cascade involved in growth hormone signaling pathway	<0.001	Interleukin signaling pathway	<0.001
Positive regulation of cytokine production	<0.001	PDGF signaling pathway	<0.001
Response to interleukin-2	<0.001	Interferon-gamma signaling pathway	<0.001
Positive regulation of T cell differentiation	<0.001	EGF receptor signaling pathway	<0.001
Positive regulation of tyrosine phosphorylation of STAT protein	<0.001	Integrin signaling pathway	<0.001
Regulation of interferon-gamma-mediated signaling pathway	<0.001	Inflammation mediated by chemokine and cytokine signaling pathway	<0.001
MAPK cascade	<0.001		
Adaptive immune response	<0.001	p53 pathway feedback loops 2	0.0025
Innate immune response	0.0014	PI3 kinase pathway	0.0027
Positive regulation of T cell proliferation	0.0022	VEGF signaling pathway	0.0045
Positive regulation of inflammatory response	0.0025	B cell activation	0.0045
Antigen receptor-mediated signaling pathway	0.0072	Ras pathway	0.0050
T cell differentiation	0.0085	T cell activation	0.0078
Inflammatory response	0.0194	Cadherin signaling pathway	0.0201
Positive regulation of antigen receptor-mediated signaling pathway	0.0227		
Transcription factor import into nucleus	0.0313		
T cell costimulation	0.0396		
*M12*		Inflammation mediated by chemokine and cytokine signaling pathway	<0.001
G-protein-coupled receptor signaling pathway	<0.001		
Chemokine-mediated signaling pathway	<0.001	Heterotrimeric G-protein signaling pathway-Gi alpha and Gs alpha-mediated pathway	0.0473
Positive regulation of cytosolic calcium ion concentration	<0.001		
Inflammatory response	<0.001		
Cell chemotaxis	<0.001		
Positive regulation of neutrophil chemotaxis	0.0136		
Response to lipopolysaccharide	0.0268		
*M13*			
Adherens junction assembly	<0.001	Integrin signaling pathway	<0.001
Phosphatidylinositol phosphorylation	0.0015	Cadherin signaling pathway	<0.001
Vesicle-mediated transport	0.0026		
Positive regulation of protein localization to nucleus	0.0043		
Actin cytoskeleton organization	0.0105		
Cell differentiation	0.0308		
*M14*			
Positive regulation of T cell activation	0.0035	T cell activation	<0.001
Interleukin-2-mediated signaling pathway	0.0065	Integrin signaling pathway	<0.001
Interleukin-4-mediated signaling pathway	0.0065	Angiogenesis	0.0032
Regulation of immune response	0.0430	Toll like receptor signaling pathway	0.0269
		VEGF signaling pathway	0.0387
		B cell activation	0.0387
		Ras pathway	0.0431

**Table 4:** Selection of DEGs in NCGS that are also modulated in human T84 cells after stimulation with anti-VP7 rotavirus peptide antibodies.

Gene symbol	Accession number	Gene title	FC NCGS PBCs	FC T84 treated cells
*Apoptosis*				
SOCS3	NM_003955	Suppressor of cytokine signaling 3	1.83	2.75
ANXA6	NM_001155	Annexin A6	1.57	2.72
SOS2	NM_006939	Son of sevenless homolog 2 (Drosophila)	1.90	1.75
DEDD	AF064605	Death effector domain containing	1.78	1.47
*Immune response*				
IFNA17	NM_021268	Interferon, alpha 17	1.59	1.56
IL6R	S72848	Interleukin 6 receptor	1.79	2.76
IRF5	NM_03264335	Interferon regulatory factor 5	1.52	1.52
CD84	AF054818	CD84 molecule	2.55	3.40
*Inflammatory response*				
IL1B	NM_000576	Interleukin 1, beta	1.52	1.80
IL24	NM_006850	Interleukin 24	2.84	2.19
IL2RA	K03122	Interleukin 2 receptor, alpha	1.86	1.48
S100A8	AW238654	S100 calcium-binding protein A8	3.65	1.86
*Cell proliferation*				
FGFR2	NM_022975	Fibroblast growth factor receptor 2	1.56	2.89
RAC2	NM_002872	Ras-related C3 botulinum toxin substrate 2	2.31	1.53
CDK2	AB012305	Cyclin-dependent kinase 2	1.63	1.78
DLG1	AL121981	Discs, large homolog 1 (Drosophila)	1.57	1.74
*Cell differentiation*				
GAS7	BC006454	Growth arrest-specific 7	−2.03	−1.90
SRD5A1	NM_001047	Steroid-5-alpha-reductase, alpha polypeptide 1	−2.53	−1.54
VAMP5	NM_006634	Vesicle-associated membrane protein 5	−1.71	−1.58
ZAK	NM_016653	Sterile alpha motif and leucine zipper containing kinase AZK	−2.02	−1.71
*Cell–cell junctions*				
VCL	NM_014000	Vinculin	−1.68	−1.56
CTNND1	NM_001331	Catenin (cadherin-associated protein), delta 1	−2.33	−1.75
CTNNA1	NM_001903	Catenin (cadherin-associated protein), alpha 1, 102 kDa	−2.49	−1.57
COL8A2	NM_005202	Collagen, type VIII, alpha 2	−1.62	−1.64
*Metalloproteases*				
ADAM8	AI814527	ADAM metallopeptidase domain 8	1.94	1.57
ADAM9	NM_003816	ADAM metallopeptidase domain 9	2.81	1.48
ADAM17	AI797833	ADAM metallopeptidase domain 17	1.51	1.56
*Receptors and signal transduction*				
IL2RA	K03122	Interleukin 2 receptor, alpha	1.86	1.48
IRF5	NM_03264335	Interferon regulatory factor 5	1.52	1.52
IL6R	S72848	Interleukin 6 receptor	1.79	2.76
*Cytoskeleton*				
FGD6	NM_018351	FYVE, RhoGEF, and pH domain containing 6	−2.40	−1.48
ABLIM3	NM_014945	Actin-binding LIM protein family, member 3	1.86	1.49
PFN2	NM_002628	Profilin 2	1.51	1.47
*Ion transport*				
SLC24A1	AF026132	Solute carrier family 24 (Na/K/Ca exchanger), member 1	1.59	1.95
SLC30A1	AI972416	Solute carrier family 30 (zinc transporter), member 1	1.94	1.55
SLC4A4	AF069510	Solute carrier family 4, NaHCO_3_ cotransporter, member 4	1.52	1.92
*EGFR signaling pathway*				
AKT2	NM_001626	v-akt murine thymoma viral oncogene homolog 2	2.36	2.19
PIK3R1	NM_181523	Phosphoinositide-3-kinase, regulatory subunit 1 (alpha)	2.76	1.54
PTPN12	S69182	Protein tyrosine phosphatase, nonreceptor type 12	2.27	1.50

**Table 5:** Genes modulated in the three datasets playing a role in selected GO BPs related to the viral infection process.

Gene symbol	Gene title	FC
*NCGS dataset*		
Viral transcription/gene expression		
RANBP2	RAN-binding protein 2	1.98
RPL27A	Ribosomal protein L27a	3.22
RPL37A	Ribosomal protein L37a	2.94
RPLP2	Ribosomal protein, large, P2	2.15
RPS10	Ribosomal protein S10	2.66
RPS11	Ribosomal protein S11	2.49
TPR	Translocated promoter region, nuclear basket protein	4.40
Response to virus		
RELA	v-rel reticuloendotheliosis viral oncogene homolog A (avian)	1.54
IKBKB	Inhibitor of kappa light polypeptide gene enhancer in B cells, kinase beta	2.69
IRF5	Interferon regulatory factor 5	1.52
IFNA17	Interferon, alpha 17	1.59
DDX3X	DEAD (Asp-Glu-Ala-Asp) box polypeptide 3, X-linked	3.22
STAT2	Signal transducer and activator of transcription 2, 113 kDa	1.59
STAT1	Signal transducer and activator of transcription 1, 91 kDa	2.73
IRF3	Interferon regulatory factor 3	1.67
DDX17	DEAD (Asp-Glu-Ala-Asp) box helicase 17	4.75
Viral life cycle		
TPR	Translocated promoter region, nuclear basket protein	4.40
ATG16L1	Autophagy-related 16-like 1 (*S. cerevisiae*)	1.87
HSP90AB1	Heat shock protein 90 kDa alpha (cytosolic), class B member 1	1.87
RANBP2	RAN-binding protein 2	1.98
DPP4	Dipeptidyl-peptidase 4	1.61
DDX6	DEAD (Asp-Glu-Ala-Asp) box helicase 6	4.70
HTATSF1	HIV-1 Tat specific factor 1	2.23
SLAMF1	Signaling lymphocytic activation molecule family member 1	1.65
T84 dataset		
Viral transcription/gene expression		
RPL27A	Ribosomal protein L27a	1.68
RPS2	Ribosomal protein S2	1.99
RPS6	Ribosomal protein S6	1.51
Response to virus		
IFIH1	Interferon induced with helicase C domain 1	1.52
IFNA7	Interferon, alpha 7	1.53
IFIT3	Interferon-induced protein with tetratricopeptide repeats 3	1.46
IFNA4	Interferon, alpha 4	1.73
IFI44	Interferon-induced protein 44	1.46
IFNGR1	Interferon gamma receptor 1	1.67
IFNA17	Interferon, alpha 17	1.56
Viral life cycle		
CTBP1	C-terminal-binding protein 1	1.58
ADRBK1	Adrenergic, beta, receptor kinase 1	1.46
HCRP1	Hepatocellular carcinoma-related HCRP1	1.61
C9Orf28	Chromosome 9 open reading frame 28	1.56
Rotavirus infection dataset		
Viral transcription/gene expression		
NUP58	Nucleoporin 58 kDa	6.38
RPS16	Ribosomal protein S16	2.10
DENR	Density-regulated protein	2.11
Response to virus		
XPR1	Xenotropic and polytropic retrovirus receptor 1	1.72
CNOT7	CCR4-NOT transcription complex subunit 7	3.54
CD40	CD40 molecule, TNF receptor superfamily member 5	2.72
ITCH	Itchy E3 ubiquitin protein ligase	2.26
ARF1	ADP-ribosylation factor 1	1.91
BCL2L11	BCL2-like 11 (apoptosis facilitator)	3.21
BCL2L1	BCL2-like 1	3.37
IKBKE	Inhibitor of kappa light polypeptide gene enhancer in B cells, kinase ɛ	1.50
DDX17	DEAD (Asp-Glu-Ala-Asp) box helicase 17	2.13
Viral life cycle		
NUP153	Nucleoporin 153 kDa	2.01
VPS37A	Vacuolar protein sorting 37 homolog A (S. cerevisiae)	1.90
XPR1	Xenotropic and polytropic retrovirus receptor 1	1.72
UBB	Ubiquitin B	1.75
ITCH	Itchy E3 ubiquitin protein ligase	2.26
NUP58	Nucleoporin 58 kDa	6.38
TNFRSF4	Tumor necrosis factor receptor superfamily, member 4	1.94
SCARB2	Scavenger receptor class B, member 2	1.96

**Table 6:** Selection of genes modulated in human T84 cells after stimulation with anti-VP7 rotavirus peptide antibodies, involved in immune response and in molecular signaling related to the viral infection process.

Gene symbol	Gene title	FC
*Immune response*		
CCR2	Chemokine (C-C motif) receptor 2	−1.48
CXCL1	Chemokine (C-X-C motif) ligand 1	1.81
CXCL13	Chemokine (C-X-C motif) ligand 13	−5.52
GATA3	GATA-binding protein 3	−6.62
TROVE2	TROVE domain family, member 2	−1.64
ICOSLG	Inducible T cell costimulator ligand	2.51
FCGR1A	Fc fragment of IgG, high affinity Ia, receptor (CD64)	2.00
FOXP3	Forkhead box P3	1.49
ULBP1	UL16-binding protein 1	−1.77
ITGA4	Integrin, alpha 4 (antigen CD49D, alpha 4 subunit of VLA-4 receptor)	1.48
CXCL9	Chemokine (C-X-C motif) ligand 9	1.59
CSF3	Colony-stimulating factor 3 (granulocyte)	1.46
IL6	Interleukin 6 (interferon, beta 2)	1.51
CD84	CD84 molecule	3.40
FCGR2B	Fc fragment of IgG, low affinity IIb, receptor (CD32)	1.77
LAT2	Linker for activation of T cells family, member 2	1.85
C7	Complement component 7	3.11
CCR1	Chemokine (C-C motif) receptor 1	3.27
CCR3	Chemokine (C-C motif) receptor 3	2.80
CFP	Complement factor properdin	2.92
IL24	Interleukin 24	2.19
IL8	Interleukin 8	1.86
CXCL10	Chemokine (C-X-C motif) ligand 10	1.82
IL1F7	Interleukin 1 family, member 7 (zeta)	−2.26
IKBKB	Inhibitor of kappa light polypeptide gene enhancer in B cells, kinase beta	−2.25
CCL11	Chemokine (C-C motif) ligand 11	1.96
*Type I interferon signaling*		
Cellular response to interferon alpha		
FCAR	Fc fragment of IgA, receptor for	2.15
Type I interferon signaling		
IFNA16	Interferon, alpha 16	1.58
STAT1	Signal transducer and activator of transcription 1, 91 kDa	−1.46
IFNA17	Interferon, alpha 17	1.56
YY1	YY1 transcription factor	−2.24
IFNA4	Interferon, alpha 4	1.73
IRF8	Interferon regulatory factor 8 interferon regulatory factor 8	−1.68
IFNA5	Interferon, alpha 5	−2.85
IRF2	Interferon regulatory factor 2	1.58
IFNA8	Interferon, alpha 8	2.23
IRF5	Interferon regulatory factor 5	1.52
IFI6	Interferon, alpha-inducible protein 6	1.56
IFNA6	Interferon, alpha 6	2.08
Positive regulation of interferon alpha production		
IRF5	Interferon regulatory factor 5	1.52
Positive regulation of interferon beta production		
DDX3X	DEAD (Asp-Glu-Ala-Asp) box polypeptide 3, X-linked	−1.49
IRF5	Interferon regulatory factor 5	1.52
Negative regulation of interferon beta production		
LILRB1	Leukocyte immunoglobulin-like receptor, subfamily B, member 1	−1.60
Positive regulation of Type I interferon production		
IFI16	Interferon, gamma-inducible protein 16	−1.68
CREBBP	CREB-binding protein (Rubinstein-Taybi syndrome)	1.51
Negative regulation of Type I interferon production		
CYLD	Cylindromatosis (turban tumor syndrome)	−3.04
*Gamma interferon signaling*		
Cellular response to Interferon Gamma signaling		
FCAR	Fc fragment of IgA, receptor for	2.15
MRC1	Mannose receptor, C type 1	2.52
SYNCRIP	Synaptotagmin-binding, cytoplasmic RNA-interacting protein	−1.69
CCL8	chemokine (C-C motif) ligand 8	1.63
Interferon gamma signaling		
STAT1	Signal transducer and activator of transcription 1, 91 kDa	−1.46
MID1	Midline 1 (Opitz/BBB syndrome)	−1.99
HLA-DRB4	Major histocompatibility complex, class II, DR beta 4	2.39
SDK1	Sidekick homolog 1 (chicken)	1.61
IFNGR1	Interferon gamma receptor 1 interferon gamma receptor 1	1.67
Negative regulation of gamma interferon production		
LILRB1	Leukocyte immunoglobulin-like receptor, subfamily B, member 1	−1.60
CD244	CD244 molecule, natural killer cell receptor 2B4	−1.69
IL10	Interleukin 10	−3.56
Positive regulation of gamma interferon production		
FOXP3	Forkhead box P3	1.49
IL1B	Interleukin 1, beta	1.80
*Toll-like receptor signaling*		
TANK	TRAF family member-associated NFKB activator	−1.91
CHUK	Conserved helix-loop-helix ubiquitous kinase	−1.72
ELK1	ELK1, member of ETS oncogene family	3.70
MAP3K8	Mitogen-activated protein kinase kinase kinase 8	−2.16
TLR6	Toll-like receptor 6	2.43
TLR1	Toll-like receptor 1	1.57
TLR7	Toll-like receptor 7	−1.64
MAP3K7	Mitogen-activated protein kinase kinase kinase 7	−1.89
LY96	Lymphocyte antigen 96	−1.81
NFKB2	Nuclear factor of kappa light polypeptide gene enhancer in B cells 2 (p49/p100)	1.54
REL	v-rel reticuloendotheliosis viral oncogene homolog (avian)	−1.82
PTGS2	Prostaglandin-endoperoxide synthase 2 (prostaglandin G/H synthase and cyclooxygenase)	1.76
TNFAIP3	Tumor necrosis factor, alpha-induced protein 3	1.73
MAP2K3	Mitogen-activated protein kinase kinase 3	1.59
IKBKB	Inhibitor of kappa light polypeptide gene enhancer in B cells, kinase beta	−2.25
TLR3	Toll-like receptor 3	−2.03
IFNB1	Interferon, beta 1, fibroblast	−1.84
IRAK3	Interleukin-1 receptor-associated kinase 3	1.70
TLR4	Toll-like receptor 4	1.46
IKBKE	Inhibitor of kappa light polypeptide gene enhancer in B cells, kinase epsilon	2.06
MAP2K2	Mitogen-activated protein kinase kinase 2	1.98
TLR2	Toll-like receptor 2	−2.30

**Table 7:** Selection of genes modulated in PBCs in course of the acute phase of rotavirus infection, involved in immune response and in molecular signaling related to the viral infection process.

Gene symbol	Gene title	FC
*Immune response*		
ADGRE3	Adhesion G protein-coupled receptor E3;ADGRE3;ortholog	−3.35
ADIPOQ	Adiponectin, C1Q and collagen domain containing	−1.60
BLK	BLK proto-oncogene, Src family tyrosine kinase	1.72
BRAF	B-Raf proto-oncogene, serine/threonine kinase	1.57
BTK	Bruton agammaglobulinemia tyrosine kinase	−1.59
C1QTNF9	C1q and tumor necrosis factor related protein 9	−1.69
CD109	CD109 molecule	−1.84
CD79B	CD79b molecule, immunoglobulin-associated beta	−1.67
CLEC7A	C-type lectin domain family 7, member A	−1.62
CMIP	c-Maf inducing protein	−3.50
CSF2RA	Colony-stimulating factor 2 receptor, alpha, low-affinity (granulocyte-macrophage)	−2.30
CXCL2	Chemokine (C-X-C motif) ligand 2	−3.76
CXCL8	Chemokine (C-X-C motif) ligand 8	−8.65
FCER1A	Fc fragment of IgE, high affinity I, receptor for; alpha polypeptide	−5.64
HCST	Hematopoietic cell signal transducer	1.85
IL18BP	Interleukin 18 binding protein	1.56
JAG1	Jagged 1	−2.06
KLRB1	Killer cell lectin-like receptor subfamily B, member 1	−5.99
MAP3K11	Mitogen-activated protein kinase kinase kinase 11	1.55
MASP1	Mannan-binding lectin serine peptidase 1	−1.50
MR1	Major histocompatibility complex, class I-related	8.43
PLEKHN1	Pleckstrin homology domain containing, family N member 1	−1.99
PPP2R2C	Protein phosphatase 2, regulatory subunit B, gamma	−1.84
PPP3CA	Protein phosphatase 3, catalytic subunit, alpha isozyme	−1.89
PSME3	Proteasome activator subunit 3	1.91
PVR	Poliovirus receptor	−1.59
STAT5B	Signal transducer and activator of transcription 5B	−2.06
TNFRSF10C	Tumor necrosis factor receptor superfamily, member 10c decoy without an intracellular domain	−1.75
TNFRSF4	Tumor necrosis factor receptor superfamily, member 4	1.94
*Type I interferon signaling*		
Positive regulation of Type I interferon production		
EP300	E1A-binding protein p300	−1.56
POLR3G	Polymerase (RNA) III (DNA directed) polypeptide G (32kD)	−1.98
CREBBP	CREB-binding protein	−1.83
LRRFIP1	Leucine rich repeat (in FLII) interacting protein 1	−2.14
SOCS1	Suppressor of cytokine signaling 1	2.32
Negative regulation of Type I interferon production		
UBB	Ubiquitin B	1.75
ITCH	Itchy E3 ubiquitin protein ligase	2.26
TAX1BP1	Tax1 (human T cell leukemia virus type I) binding protein 1	−4.01
Negative regulation of Type I interferon pathway		
PTPN2	Protein tyrosine phosphatase, nonreceptor type 2	2.10
Positive regulation of interferon beta production		
ZBTB20	Zinc finger and BTB domain containing 20	4.27
Negative regulation of interferon Beta production		
PTPRS	Protein tyrosine phosphatase, receptor type, S	−2.00
CACTIN	Cactin, spliceosome C complex subunit	−2.53
Cellular response to interferon alpha		
TPR	Translocated promoter region, nuclear basket protein	−2.55
Negative regulation of interferon alpha production		
PTPRS	Protein tyrosine phosphatase, receptor type, S	−2.00
Type I interferon signaling pathway		
JAK1	Janus kinase 1	1.79
IFI27	Interferon, alpha-inducible protein 27	75.26
IFI27L2	Interferon, alpha-inducible protein 27-like 2	−1.59
IKBKE	Inhibitor of kappa light polypeptide gene enhancer in B cells, kinase epsilon	1.50
TPR	Translocated promoter region, nuclear basket protein	−2.55
Positive regulation of Type I interferon pathway		
MME	Membrane metallo-endopeptidase	−5.54
*Gamma interferon signaling*		
JAK1	Janus kinase 1	1.79
HLADQB1	Major histocompatibility complex, class II, DQ beta 1	−36.43
HLADQA1	Major histocompatibility complex, class II, DQ alpha 1	−37.95
PIAS3	Protein inhibitor of activated STAT 3	2.11
HLADRB1	Major histocompatibility complex, class II, DR beta 1	−10.91
MAPK8	Mitogen-activated protein kinase 8	−2.28
MAPK1	Mitogen-activated protein kinase 1	−2.23
Regulation of interferon gamma signaling pathway		
PTPN2	Protein tyrosine phosphatase, nonreceptor type 2	2.10
Positive regulation of interferon gamma production		
PDE4B	Phosphodiesterase 4B, cAMP-specific	−1.77
ZFPM1	Zinc finger protein, FOG family member 1	1.75
Negative regulation of interferon gamma production		
HLADRB1	Major histocompatibility complex, class II, DR beta 1	−10.91
RARA	Retinoic acid receptor, alpha	−7.60
FOXP3	Forkhead box P3	−2.14
Cellular response to interferon gamma		
SLC26A6	Solute carrier family 26 (anion exchanger), member 6	1.95
DAPK3	Death-associated protein kinase 3	−1.62
CD40	CD40 molecule, TNF receptor superfamily member 5	2.72
MEFV	Mediterranean fever	−4.83
SNCA	Synuclein alpha	8.62
MRC1	Mannose receptor, C type 1	−1.93
*Toll-like receptors signaling pathway*		
TANK	TRAF family member-associated NFKB activator	−1.91
NFKBIA	Nuclear factor of kappa light polypeptide gene enhancer in B cells inhibitor, alpha	−1.76
MAPK8	Mitogen-activated protein kinase 8	−2.28
IKBKE	Inhibitor of kappa light polypeptide gene enhancer in B cells, kinase epsilon	1.50
MAPK1	Mitogen-activated protein kinase 1	−2.23
JUN	jun proto-oncogene	−1.91

## Supplementary Materials

Supplementary 1 Additional Table 1: genes modulated in NGCS samples versus healthy controls.

Supplementary 2 Additional Table 2: genes modulated in the “Rotavirus infection” dataset.

Supplementary 3 Additional Table 3: GO terms containing the words “virus” and “viral” to which are associated genes modulated in the three datasets.
